# Objective and Subjective Cognitive Function, and Relations With Quality of Life and Psychological Distress

**DOI:** 10.1093/geroni/igaa057.982

**Published:** 2020-12-16

**Authors:** Hallie Nuzum, Katherine Dorociak, Shirit Kamil-Rosenberg, Peter Louras, Mandana Mostofi, J Kaci Fairchild

**Affiliations:** 1 VA Palo Alto Healthcare System, Palo Alto, California, United States; 2 Palo Alto University, Palo Alto, California, United States

## Abstract

Objective and subjective cognitive function have been associated with decreased quality of life and increased psychological distress in older adults. The present study examined relations of objective and subjective cognition with quality-of-life and mental-health outcomes in individuals with amnestic mild cognitive impairment (aMCI). The sample included 98 older adults with aMCI (92.5% male, age = 70.9±9.2 years). Measures included objective cognition (i.e., attention, memory, language, visuospatial abilities, processing speed, executive function, and overall), subjective memory (Multifactorial Memory Questionnaire [MMQ]), quality of life (Dementia Quality of Life [DQoL]), and mental health (Geriatric Depression Scale, Geriatric Anxiety Inventory, and Penn State Worry Questionnaire). Objective and subjective cognition were weakly correlated (range |r| = .00–.23). Objective cognitive measures were largely uncorrelated with quality of life or mental health, with only two significant (p < .05) correlations between Processing Speed and Worry (r = -.24), and Overall Cognition and DQoL Aesthetics (r = .20). Subjective cognition was more strongly correlated with quality of life, including significant (p < .01) correlations between MMQ Abilities and DQoL Negative Affect (r = -.38), and MMQ Contentment and DQoL Positive Affect (r = .28). Additionally, MMQ Contentment and Abilities were significantly (p < .01) negatively correlated with all three mental-health outcomes (range |r| = .28–.43). This study demonstrated that subjective memory, particularly affect and self-appraisal regarding one’s memory capabilities, is more closely related to quality-of-life and mental-health outcomes than objective cognitive performance in an aMCI sample, and, therefore, may represent important targets for intervention.

